# Molecular farming expression of recombinant fusion proteins applied to skincare strategies

**DOI:** 10.7717/peerj.17957

**Published:** 2024-09-18

**Authors:** Guangdong Yu, Wengang Zhao, Yunpeng Wang, Nuo Xu

**Affiliations:** 1 College of Life and Environmental Sciences, Wenzhou University, Wen Zhou, China; 2 Jilin Academy of Agricultural Sciences, Northeast Innovation Center of China Agricultural Science and Technology, Ji Lin, China

**Keywords:** Molecular farming, Skincare, Arabidopsis, Transdermal peptide, Epidermal growth factor

## Abstract

This review discusses the current research progress in molecular farming technology in the field of skincare, with an emphasis on molecular farming expression strategies. The strategies of transdermal drug delivery and their advantages are also highlighted. The expression of cosmetically relevant fused proteins has become an important way to enhance the efficacy of the proteins. Therefore, we also discuss the feasibility and strategies for expressing fusion proteins in *A. thaliana*, specifically the fusion of Epidermal growth factor (EGF) to a cell-penetrating peptide (CPP), in which the production can be greatly enhanced *via* plant expression systems since these systems offer higher biosecurity, flexibility, and expansibility than prokaryotic, animal and mammalian expression systems. While the fusion of EGF to CCP can enhance its transdermal ability, the effects of the fusion protein on skin repair, melasma, whitening, and anti-aging are poorly explored. Beyond this, fusing proteins with transdermal peptides presents multiple possibilities for the development of tissue repair and regeneration therapeutics, as well as cosmetics and beauty products. As certain plant extracts are known to contain proteins beneficial for skin health, the expression of these proteins in plant systems will better maintain their integrity and biological activities, thereby facilitating the development of more effective skincare products.

## Molecular farming for skincare research

Molecular farming is an interdisciplinary subject of agronomy, engineering, and medicine, as it can be widely used in industry, environmental protection, and health industries. Molecular farming, also known as a plant bioreactor, utilizes gene editing technology to enable the expression of pharmacologically important proteins in plant models (Kowalczyk et al., 2022). There are three types of transformation. The target gene in the plant expression vector is transformed into the plant genome by *Agrobacterium tumefaciens* to yield a stable heredity, called stable nuclear transformation. Alternatively, transient expressions based on agroinfiltration or virus-based vectors have been developed to complement transgenic plants that offer rapid and high-level protein expression within a few days. Alternately, the target gene was inserted into the chloroplast genome, and the foreign protein was expressed in the chloroplast (Krenek et al., 2015; Liu et al., 2023; Shanmugaraj, Bulaon & Phoolcharoen, 2020). Owing to its advantages and rapid development, molecular farming has attracted global attention from researchers and industries, including skincare research and industry (Shanmugaraj, Bulaon & Phoolcharoen, 2020). Advances in biotechnology have led to the establishment of efficient plant expression systems, most of which have been extensively studied. Currently, plants such as *Nicotiana tabacum L*, *Brassica napus*, *Solanum tuberosum L*, *Solanum lycopersicum L*, *Glycine max*, *Arabidopsis thaliana L*, *Zea mays L*, and *Oryza sativa* seeds have been used to express foreign recombinant proteins, thus earning plant bioreactors the name “chemical factories.” Recent use of plant bioreactors hinges on the expression of biological agents with potential commercial value, such as vaccine antigens, antibodies, nutritional supplements, and industrial enzymes (Kumar, Singh & Singh, 2022; Yang et al., 2021). With the commercialization of the recombinant glucosidase drug, the first commercialized bioactive agent expressed in plant cells for the treatment of Gaucher’s disease, it is safe to say that the use of plant bioreactors has entered a new era (Kowalczyk et al., 2022). Other proteins that have been expressed in plant bioreactors include human serum albumin (HSA), expressed in *O. sativa*. Using crystallography, the plant bioreactor-expressed HSA was confirmed to be structurally similar to the plasma HSA, suggesting that they share the same biological activity (Kuo et al., 2013). In veterinary medicine, the subunit vaccine of bovine viral diarrhea virus (BVDV), a serious cause of global economic losses in farm animals, has been expressed in modified alfalfa crops and is shown to be highly effective (Aguirreburualde et al., 2013). The human insulin-like growth factor binding protein-3 (Higfbp-3), a multifunctional protein, is another protein that has been expressed in plants, specifically *N. tabacum*. Interestingly, the protein was expressed in *N. tabacum* seeds using the signal peptide (SP) and c-terminal tetrapeptide AFVY of phaseolin. Analysis of expression showed that recombinant Higfp-3 accumulated in *N. tabacum* seed with a high yield (Cheung et al., 2009). Multiple therapeutic proteins synthesized in plants are either at the preclinical or clinical trials stage with the potential of achieving commercialization. For example, in the area of skin health, multiple studies have shown the applicability of molecular farming in skin health research and skin care product development. Nazeri, Aram & Niazi (2023) used *N. tabacum* to express hyaluronic acid (HA) and found that plants can produce the precursor HA but not the mature HA as they lack hyaluronic acid synthase. To overcome this limitation, these authors successfully transfected the human hyaluronate synthase two gene into *N. tabacum* and confirmed its retention of function, thus enabling the production of mature HA in plants (Nazeri, Aram & Niazi, 2023). Other genes relevant to skincare that have been expressed in plants include superoxide dismutase (SOD) and basic fibroblast growth factor (bFGF). Previously, SOD was mainly derived from animal tissues. However, in the past decade, functional SOD with potential application across various biological fields has been obtained from plant-based expression systems (Che et al., 2020; Ren et al., 2018). Ding et al. (2006) expressed bioactive bFGF in soybeans and showed that the recombinant protein can retain its proliferative function. These findings confirm the feasibility of plants as natural bioreactors for the production of cytokines and growth factors relevant to skincare.

## Review methodology

The data presented herein were acquired following a rigorous search from several well-established academic search engines and specific journal databases. These included Google eScholar, PubMed, Web of Science, Baidu Academic, and Microsoft Academic Search. Google eScholar and PubMed served as the main search tools, accounting for more than half of the used literature. To optimize and find the relevant literature, we used the boolean search method with the following keywords: Arabidopsis and transgenic, molecular agriculture, molecular farming, plant bioreactor, transdermal peptide, cytokine and transdermal peptide, molecular agriculture and pharmaceutical protein, cosmetics and plant bioreactor, and molecular agriculture and cytokines. Thereafter, relevant reports were acquired, studied, and assessed for suitability to the topic and content of the review, thus ensuring the quality and accuracy of interpretations of the findings and conclusions presented herein.

## Commercial strategies for expression of foreign proteins using molecular agriculture

### Strategies for increasing bioaccumulation in molecular agriculture

Higher expression yields of foreign proteins in plants remain a major constraint to the commercialization of these proteins, especially skin health-relevant proteins. As such, there is a need for enhanced plant bioreactors or improved expression strategies. Several strategies have been employed to optimize plant bioreactors for the synthesis of pharmaceutical proteins. These strategies include the mode at which the target genes are introduced into the plants, the type of promoters used, the enhancement of specific transcription and the stability of the transcripts, the enhancement of the translation of foreign proteins, and the accumulation of foreign proteins in specific plant parts. Specific modification strategies vary depending on the choice of plant used to express the protein. For example, to increase collagen 1 expression in transgenic *N. tabacum*, Ruggiero et al. (2000) used expression vectors containing enhanced 35S promoter and 35S terminator of the cauliflower mosaic virus and transfected them into the *N. tabacum* leaf disc using a recombinant *Aspergillus*. This resulted in a yield of about 3 g of purified recombinant collagen per 100 kg of powdery plant (Ruggiero et al., 2000). Stein et al. (2009) also expressed recombinant heterotrimeric collagen type I (RHCOL1), human prolyl-4-hydroxylase (P4H), and lysyl hydroxylase 3 (LH3) together in *N. tabacum*. P4H and LH3 serve as key posttranslational modifiers of RHCOL1 to produce a thermally stable triple helix protein. This expression strategy reported a yield of two percent of the total soluble protein extracted from the plants (Stein et al., 2009). Using the Shine-Dalgarno sequence (SD) to express human serum albumin (HSA) in transgenic chloroplasts. Although SD normally promotes high expression of the transgene, the amount of HSA obtained in this study accounts for only 0.02 percent of the total protein. However, the expression of HSA was increased by 500-fold, accounting for 11.1 percent of the total protein, when the chloroplast untranslated region (UTR) was used to modify the regulatory sequence of HSA (Fernández-San Millán et al., 2003). Stable nuclear transformation is the prerequisite for the expression of foreign proteins in the whole plant. Stable nuclear transformation is a traditional strategy of plant gene manipulation for the production of recombinant proteins. In this strategy, the target gene can be stably integrated into the plant genome, and the recombinant protein can be produced continuously throughout the plant (Shanmugaraj, Bulaon & Phoolcharoen, 2020; Bhoria et al., 2022). Expression of the recombinant protein in the whole plant can greatly increase the accumulation of exogenous protein. Li et al. (2006) investigated the expression of the cholera toxin B subunit and human insulin B chain, each as a fusion protein in transgenic *N. tabacum* plants, and achieved a protein yield of 0.11 percent of total protein in the leaves. Optimizing the codon and regulatory elements and suppressing gene silencing can also improve the expression of proteins, as demonstrated by the high level of insulin expressed (Bhoria et al., 2022).

Taken together, the different strategies have different effects on the expression of foreign proteins in plant expression systems, making them critical to the success of plant bioreactors. Therefore, enhanced strategies will play a vital role in solidifying the use of molecular agriculture in the development of skin health and skin care products.

### Strategies for improving the self-efficacy of molecular agricultural fusion proteins

The skin is the largest organ of the human body, and it serves as the first barrier of defense for the body, regulating the entry and release of substances (Hänel et al., 2013). The barrier function of the skin also restricts the penetration of most externally applied macromolecules. The skin barrier allows the penetration of molecules with a molecular weight of approximately 500 Da and below (Bos & Meinardi, 2000). However, the majority of dermal therapeutics have a molecular weight higher than 500 D (Zhang et al., 2021). For example, EGF has a molecular weight of about 6,045 Da (Adamson & Rees, 1981), and this impedes its efficiency and often requires large-scale administration to achieve any therapeutic effect. Large-scale administration may result in undesirable outcomes, such as irritation or allergic reactions. As such, skincare products require effective delivery methods to ensure their penetration into the transdermal section of the skin.

The efficacy of the skincare products can be improved by using an appropriate transdermal delivery system (Zhang et al., 2021). Transdermal drug delivery has emerged as a preferred delivery method in skincare treatment as the drugs can be applied to the intact skin. There are various approaches to transdermal drug delivery, and these can be tailored to the nature and type of skincare products. Transdermal delivery methods are mainly divided into physical methods, chemical methods, and biological methods. Physical methods include iontophoresis, ultrasonic treatment, electroporation, and the use of microneedles. These methods enable the drug to quickly penetrate the skin barrier, but they are known to cause discomfort to the users (Bok et al., 2023; Dermol-Černe, Pirc & Miklavčič, 2020; Hong et al., 2018). Chemical methods involve modifying the chemical structure of the skincare product or adding chemicals to form microcarriers or capsules that carry the skincare products across the skin (Akram et al., 2022; Damiani & Puglia, 2019). While chemical methods do not cause discomfort, such as pain, to users, the modification is likely to change the physical and chemical properties of the products. Furthermore, some chemicals may cause irritation or allergic reactions to the skin or abrogate the skin barrier function, making it unsafe for the user. For example, topically applied ethanol acts as a skin penetration enhancer and may facilitate the transdermal absorption of xenobiotics. Ethanol use is associated with skin irritation or contact dermatitis, especially in humans with an aldehyde dehydrogenase (ALDH) deficiency (Lachenmeier, 2008). Azone,1-dodecylhexahydro-2H-azepin-2-one, N-dodecyl-2-pyrrolidinone, N-dodecyl-2-piperidinone, N-dodecyl-N-(2-methoxyethyl)acetamide and 2-(1-nonyl)1,3-dioxolane were found to cause severe irritation to the skin (Phillips & Michniak, 1995). In biological methods, penetrating peptides are first screened for suitability, and the suitable peptide is then fused to the protein of interest. This increases the ability of the protein to penetrate the skin barrier without altering its function (Chablani & Singh, 2022). Biological transdermal methods have high biosafety to reduce the risk of harming the skin barrier or inducing undesirable reactions.

In summary, the biological, transdermal approach is the ideal choice as it can promote the effectiveness of the protein through fusion with cell penetrating peptides (CPPs). Recombinant DNA technology has enabled the synthesis of fusion proteins with multi-functional properties. By fusing two or more proteins through a fusion protein linker, the fused protein acquires synergetic or enhanced functions (Chen, Zaro & Shen, 2013). Protein fusion technology is used to facilitate protein purification, protein detection, or improve protein expression levels (Terpe, 2003). In protein purification or detection, fusion does not result in the alteration of protein function or activity. For example, the fusion of green fluorescent protein (GFP) with constitutively photomorphogenic 1 (COP1) does not alter its structure, activity, or localization (Von Arnim, Deng & Stacey, 1998). GFP has also been used to track the distribution of nuclear pore complexes (NPCs) components in real-time throughout the cell cycle, as well as for high-resolution immune Scanning electron microscopy (SEM) labeling to determine localization at the nano-level (Drummond & Allen, 2008). Histidine (His) tagging, where a sequence of short peptides composed of His is fused to the N- or C-terminus of the target protein, is another fusion method widely used for protein purification and detection. His-tag-fused proteins expressed in bacteria, yeast, insect cells, or mammalian cells still retain their native function of the protein (Lee et al., 2017). Other fusion peptides may increase the solubility and secretion of the target protein. For example, maltose-binding protein acid (Mbp) significantly enhances the solubility, secretion, and purification of target proteins expressed in *E. coli* (Malik, 2016; Ki & Pack, 2020). Other known fusion protein tags include Arg-tag, calmodulin-binding peptide, cellulose-binding domain, Disulfide bond formation protein A(DsbA), c-myc-tag, glutathione S-transferase, natural His affinity tag (HAT-tag), N-utilization substance A (NusA), human ribonuclease 1 (human S-tag), streptavidin-binding peptide (SBP-tag), streptavidin tag (Strep-tag), and thioredoxin. These tags play an important role in different fields because of their various functions.

Cell-penetrating peptides (CPPs), which generally consist of 5-30 amino acids, are another group of functional tags that promote skin barrier penetration. The special ability of CPPs allows them to be used in a variety of industrial applications (Shin et al., 2022). CPP is a powerful tool for biological research, drug development, and the transdermal delivery of cosmetics. For example, CPPs such as the transdermal peptide TAT have been used to deliver siRNA therapeutics for skin diseases and improve skin characteristics. TAT can significantly improve the rate of siRNA transdermal delivery (Uchida et al., 2011). SPACE, another transdermal peptide, has been used for the delivery of cyclosporin A in the treatment of a variety of skin diseases (Chen et al., 2015). CPPs have also been used to deliver growth factors. For example, the hydrophobic peptide macromolecular transduction domain 1067 (MTD 1067) has been shown to improve Ghrp-6, insulin-like growth factor I (des (1-3) IGF-I), and platelet-derived growth factor BB (PDGF-BB) transdermal delivery by 4.4, 18.8, and 32.9 times, respectively, without causing cytotoxicity when compared with the control (Shin et al., 2022). CPPs can be co-expressed with target proteins in plant bioreactors, leading to fusion proteins with enhanced transdermal activity. CPPs have recently been shown to be effective at overcoming the selective permeability of the cell membrane. Thus, the fusion of CPPs with bioactive macromolecules can increase their ability to penetrate the cell membrane, making them a preferred transdermal delivery system (Pujals et al., 2006). To date, several CPPs have been identified and used in the transdermal delivery of biomacromolecules (Lipinski et al., 1997). Among these peptides, the mechanisms of transdermal peptide 1 (TD-1) and translocating peptide 1 (TP1) have been substantially investigated. TD1 was identified by high-throughput screening and has since been explored in terms of its skin penetration ability. Ruan et al. (2014) expressed human epidermal growth factor (hEGF) fused with TD1 in the yeast system and showed that the fusion had a higher skin penetration potential, thus laying a foundation for the application of TD1 as a transdermal delivery vector. The structure and transdermal mechanism of TP1 have been explored by Muñoz-Gacitúa, Guzman & Weiss-López (2022) using nuclear magnetic and isotope labeling methods, and the peptide has been evaluated for its delivery of antitumor drugs. For example, the delivery of carbataxel by TP1 was shown to have enhanced the treatment of prostate and breast cancer (Park et al., 2021). The promising effects of CPPs in transdermal delivery suggest that they are poised to become a preferred bio-transdermal method for skincare products.

Taken together, the expression of fusion proteins in plant expression systems can be modified to enhance their transdermal potential and the properties of the target proteins. This may serve as an important prerequisite for the commercialization of plant expression systems as well as their products.

### Strategies for improving the self-safety of molecular agroprotein fusion proteins

Prokaryotic expression systems are traditional foreign protein expression systems. Their outer membrane contains lipopolysaccharide (LPS), which can cause adverse toxic effects and restrict the application of recombinant proteins in various fields (Shahar et al., 2022). Prokaryotic expression systems can increase the cost and steps of downstream purification of foreign proteins. The production of exogenous protein by a plant expression system can effectively avoid the contamination of endotoxin, simplify the steps of downstream protein purification, and guarantee the safety of the treatment for human diseases (Shahar et al., 2023). In order to ensure the safety of the products, suitable host plants should be selected to express different special foreign proteins. Plants have already been used to express cytokines, hyaluronic acid, and collagen. The first plant transgenic system was based on *N. tabacum*, whereby the gene encoding a foreign protein was introduced into the plant *via Agrobacterium tumefaciens*. Although this system was found to improve the expression of the target protein, *N. tabacum* contains toxic alkaloids, and a small amount of these alkaloids can stimulate the central nervous system and raise blood pressure, whereas a large amount can inhibit the central nervous system, with potential consequences of heart paralysis and death. For example, a few milligrams of nicotine can cause headaches, vomiting, confusion and other poisoning symptoms. In a 3D human skin model (EpiDerm), nicotine can also affect protein composition and damage organelles affecting mitochondrial and peroxisome ROS homeostasis. These changes may exacerbate skin infections, inhibit wound healing, and cause oxidative damage to skin cells (Pozuelos et al., 2022). Thus, in terms of safety, *N. tabacum* is not suitable for the production of skincare products. More ideal plants for such purposes are *A. thaliana*, legumes, fruits, and vegetables.

## Application of cytokine expression in molecular agriculture

Molecular farming can be applied to the study and application of recombinant protein expression, and it has been demonstrated for proteins such as cytokines and growth factors. Molecular farming can also be used to assess the applicability of various expression methods. Plants have several advantages that make them efficient bioreactors. First, their offspring can stably inherit the transfected genes to ensure the continuous production of the recombinant proteins. This also allows the acquisition of plants already expressing the desired protein, thus reducing the production cost and providing the prerequisite basis for commercialization (Ma, Drake & Christou, 2003). Second, plants have functional, post-translational modification machinery (Huang & McDonald, 2012), which enables the processing of recombinant proteins to ensure the retention of structure and function. Third, unlike other expression systems, plant expression systems do not require heat sources, and the purified proteins are free from endotoxins, or other sensitizing substances. As such, these expression systems reduce the potential damage of the recombinant proteins and are likely to cause less sensitization and adverse reactions in people who use the final products (Moustafa, Makhzoum & Trémouillaux-Guiller, 2016). Fourth, except for foreign proteins, the extraction of the whole plant contains a lot of antioxidants, vitamins, and antibacterial substances, which can be easily separated from the plant (Sitarek et al., 2020; Hoang, Moon & Lee, 2021; Marchev & Georgiev, 2020). The proper mix of exogenous protein and plant derivatives plays a synergistic role in skincare (Miliauskas, Venskutonis & Van Beek, 2004). Not only can it promote the full utilization of raw materials, but it is also in line with the current demand for skincare raw materials from the pure natural concept (Hoang, Moon & Lee, 2021).

To date, multiple studies have shown the feasibility of expressing skincare-relevant proteins in plant bioreactors. Proteins that have been successfully expressed in plants include aFGF, bFGF, FGF21, KGF-2, and EGF and the actual plants used to express these proteins include *A. thaliana*, *S. lycopersicum*, *N. tabacum*, and *Carthamus tinctorius Linn*. aFGF, bFGF, KGF-2, EGF, and among others, are proven to delay aging (Mehta & Fitzpatrick, 2007; Hou et al., 2020), promote whitening (Smit, Vicanova & Pavel, 2009), remove acne and other spots, moisturize, as well as eliminate wrinkles, and brighten the skin. Additionally, some of these growth factors have been demonstrated to enhance skin regeneration and reduce scar formation (Bloemen et al., 2009; Dolivo, 2022). These studies illustrated the potential prospect of the use of plants as bioreactors in the field of skincare. Thus, how to make plant-derived proteins reflect higher application value in skin care has become an urgent problem to be solved. Studies have shown that the incorporation of the expression of cytokines and growth factors with beneficial effects on skin health in different plant parts may confer synergetic effects. Specific plant parts, such as roots, leaves, and fruits/seeds, are rich in anthocyanins, vitamin E, and antioxidants, and thus they have been utilized in multiple skincare products (Jo et al., 2020; Michalak, 2022). The expression of recombinant proteins in plant parts containing high levels of antioxidants can help increase skincare efficacy when applying into products. Another approach is the fusion protein strategy to enhance their transdermal properties (Bolhassani, Jafarzade & Mardani, 2017). Oil-body cytokine fusion can also significantly improve the transdermal rate of the cytokine. The permeability mechanism of oil body may be similar to that of liposomes. The drugs carried by liposomes show a strong ability to penetrate through the skin. Qiang et al. (2018) studied the oil body carrying oleosin-hEGF-hEGF and showed that it can rapidly penetrate the skin tissue and the amount of absorption is higher than EGF not fused to oil body. Zhao et al. (2015) used *A. thaliana* to express aFGF fused with oil body, which were specifically stored in the seeds, and subsequent toxicological tests showed that the fusion protein has no obvious toxicity and would not cause any irritation or skin allergy. Suggestively, this could allow the expression of pharmacologically beneficial proteins in the desirable plant parts or the avoidance of the expression of these proteins in certain plant parts. This can lead to increased reusability and value of plant bioreactors. Coupled with the general preference for naturally derived skincare products, molecular farming undoubtedly holds unique advantages over conventional methods used to synthesize proteins in the skincare industry.

## Feasibility of molecular farming synthesis of tdp1 fusion egf protein

Epidermal growth factor (EGF) is a small-molecule polypeptide composed of 53 amino acid residues with multiple functions (Boonstra et al., 1995). EGF has several beneficial effects on the skin and has been used for various purposes in skincare. For example, recombinant human epidermal growth factor (rhEGF) is used in anti-acne creams. Using split-face randomized double-blind trials, rhEGF has been demonstrated to significantly improve acne clearance with little to no side effects (Kim et al., 2014a). Aside from treating acne, EGF has also been used to treat skin melasma and is known to be non-invasive (Lyons, Stoll & Moy, 2018). Additionally, EGF is known to be an anti-aging factor, and its topical application reduces wrinkles while increasing dermal collagen in multiple clinical studies (Ha et al., 2017; Yu & Driscoll, 2011; Kim et al., 2014b). EGF is also known to accelerate wound healing and reduce scarring, and as such, it is widely used post-surgery, especially in reconstruction and minimally invasive facial plastic surgery (Ratanapokasatit & Sirithanabadeekul, 2022). Evidently, EGF has a wide range of applications in skincare. However, the lack of effective transdermal properties of EGF reduces the efficacy of the EGF products. Thus, the development of improved transdermal EGF delivery methods remains a major challenge for its future application in skin health (Shin et al., 2023). To resolve this, recent studies have fused EGF with TD1 (a CPP), conferring improved dermal penetration and enhanced efficacy for the TD1-EGF product (Ruan et al., 2013). However, TD1-EGF has been largely expressed in yeast or other prokaryotic systems (Ruan et al., 2014). The expression of TD1-EGF in plants may confer additional benefits mentioned above, especially when expressed in plants used as raw materials for skincare products.

A potential model plant for the expression of fused EGF is*A. thaliana*, as it has a high infection rate compared to other plants. Additionally, *A. thaliana* has a strong adaptability and fast growth rate, a large biomass, and a high content of anthocyanins, which may have synergistic effects on the skin (Zheng et al., 2021). Furthermore, *A. thaliana* extracts are used as raw materials or ingredients in multiple cosmetics. EGF has been expressed in various plant systems, including *A. thaliana*, *S. tuberosum*, *N. tabacum* and *O. sativa* (Thomas & Walmsley, 2014), but there has been no investigation into the expression of TD1-EGF. A study by Qiang et al. (2020) expressed double-EGF fused with olesion in *A. thaliana* and explored the quantity and quality of the resultant olesion-hegF-Hegf protein. The recombinant protein was found to retain the bioactivity of EGF and was more permeable compared to the control. The replication of a similar recombinant gene design may yield a superior-performing TD1-EGF. Jin et al. (2014) explored the transdermal efficacy of hEGF fused with either a single- or double-TD1 and showed that the double-TD1 fused EGF is at least five times more efficient than the control compared with just 3 $\times$ more efficient in the case of the single-TD1-fused EGF. This suggests that the higher transdermal permeability of double-TD1-fused EGF may improve the therapeutic outcome and lead to wider medical applications.

In order to ensure the stable expression of TD1 in plants, we will optimize the preference of the TD1 codon and change part of its amino acid residues through point mutation to obtain the TD1 mutant transdermal peptide TDP1, which is fused to the EGF gene that is also subjected to codon optimization. In the preliminary design, a flexible linker is inserted between the two protein molecules to prevent the structure of the two protein molecules from affecting the function of the fusion protein (Chen, Zaro & Shen, 2013). In future studies, the feasibility of TDP1-EGF expression in a whole plant system will be tested in *A. thaliana*. The transdermal ability and biological activity of TDP1-EGF will also be evaluated with references to skincare, and hopefully, this will provide a new application model of plant bioreactor in the field of skin healthcare products for exploring new ideas for the development of natural skincare raw materials.

## Outlook

Transdermal delivery is an attractive route for the administration of drugs because of its high acceptance among patients and avoidance of the first-pass hepatic metabolism. In the route of transdermal drug delivery, the availability of CPPs has led to improved efficacy of the drugs and broaden the selectivity of the drugs that can be used. In the field of skincare, epidermal growth factor (EGF) is widely recognized for its various functions, which include whitening and anti-aging effects, accelerating wound healing, and minimizing scar formation. To enhance the transdermal absorption of EGF, a transdermal peptide called TDP1 was fused to the C-terminus of EGF to produce a fusion protein known as TDP1-EGF, which can then be expressed in a plant bioreactor.

Research on plant bioreactors has been going on for over four decades. Initially, plants were used to express antibodies, vaccines, and medicinal proteins. Over time, the advantages and disadvantages of using plants as bioreactors have become clear. More and more growth factors are produced by plants and used in the field of skin care. Today, we understand that it is not necessary to express foreign proteins in specific plant tissues to improve yield. The selection of a suitable plant expression vector and transformation mode can significantly improve the bioaccumulation of the fusion protein TDP1-EGF.

For example, inflorescence transformation can integrate the target gene into the plant genome and stabilize inheritance and whole-plant expression. In addition, selecting appropriate plant expression vectors that contain an enhanced 35S promoter and 35S terminator will increase the accumulation of the fusion protein TDP1-EGF. Furthermore, it has become clear that plants are not suitable bioreactors for expressing proteins that require high purity, such as antibodies, due to the potential difficulty of separation and purification. Despite this, plants are still being explored for the expression of vaccine antigens. Indeed, plant bioreactors have a great potential for applications in various fields of medicine, and with further development, the challenge of purification will be overcome. Here, we selected two fusion protein tags, His-tag and TDP1, which not only have overcome the difficulties of downstream purification but also the difficulties of transdermal drug delivery. Advancements in the use of plant bioreactors have enabled their use in skincare product research and development. The use of plant bioreactors in skincare product development fulfills the need for effective products as well as the desire for plant-derived products. In addition, plants are considered an environmentally friendly source of raw materials, and this will ease concerns about the sources of the raw materials used in industry and research. Thus, plant bioreactors are more suitable for direct application of the expressed proteins. In order to improve the safety of the fusion protein TDP1-EGF, we expressed TDP1-EGF in Arabidopsis, because Arabidopsis has a simple biomass and a short growth cycle, and it contains anthocyanins and other by-metabolites that are conducive to skincare. Moreover, there are presently few skincare products on the market that exploit the synergetic effects of recombinant proteins and active substances in plants. Therefore, TDP1-EGF is bound to become more competitive and popular, especially in the cosmetic industry. While molecular farming has a broad prospect in the field of skincare products, there is a need to further our current understanding of the use of plants as bioreactors and how to scale up their production for the skincare market.

## Additional Information and Declarations

### Data Availability

The following information was supplied regarding data availability: This is a literature review.
